# Successful endovascular thrombectomy with the ClotTriever System for acute subclavian vein thrombosis in venous thoracic outlet syndrome

**DOI:** 10.1186/s42155-023-00378-7

**Published:** 2023-06-07

**Authors:** Andrea Discalzi, Valentina Cignini, Fernanda Ciferri, Floriana Nardelli, Denis Rossato, Marco Calandri, Paolo Fonio

**Affiliations:** Department of Surgical Sciences, Radiology Unit, University of Turin, Molinette Hospital, Via Genova 3, 10126 Turin, Italy

**Keywords:** Thoracic outlet syndrome, Upper extremity, Deep vein thrombosis, Mechanical thrombectomy, ClotTriever

## Abstract

**Background:**

The case describes a successful endovascular thrombectomy using the ClotTriever System for an acute subclavian thrombosis in venous thoracic outlet syndrome. To the best of our knowledge, this is the first case report on use of Inari ClotTriever for acute upper extremity deep venous thrombosis due to venous thoracic outlet syndrome. The rapid technical and clinical success of our intervention may be an interesting cue for interventional radiologist colleagues.

**Case presentation:**

Upper extremity deep vein thrombosis in the setting of venous thoracic outlet syndrome generally occurs in young adults after excessive arm activity and can sometimes be managed with anticoagulation. In this case, a 29-year-old male diagnosed with acute effort-induced thrombosis of the left subclavian vein and persistent symptoms following low-molecular-weight heparin therapy underwent mechanical thrombectomy. Successful thrombectomy was completed with > 90% thrombus burden reduction and no complication. The patient experienced immediate symptom relief and vein patency was confirmed via imaging 3 months post procedure.

**Conclusions:**

Mechanical thrombectomy is a promising treatment technique for thrombosis associated with venous thoracic outlet syndrome.

**Supplementary Information:**

The online version contains supplementary material available at 10.1186/s42155-023-00378-7.

## Introduction

Venous thoracic outlet syndrome (VTOS) is a rare condition resulting from compression of the subclavian vein (SV), most commonly at the costoclavicular junction [1]. SV compression can be intermittent or result in deep vein thrombosis (DVT) occlusion [1]. Upper extremity DVT in the setting of VTOS typically occurs in younger adults after repetitive excessive arm activity and can be referred to as effort thrombosis or Paget-Schroetter syndrome [1–3]. Characteristic symptoms of effort thrombosis occur due to venous obstruction and include sudden arm swelling, cyanosis, pain, and mild paresthesia. Accurate diagnosis and prompt treatment are the most important predictors of patient outcomes [2, 3].

Standard treatment of upper extremity DVT is anticoagulation, while catheter-directed thrombolysis (CDT) can be considered in patients with severe symptoms and low bleeding risk who are likely to benefit from thrombolysis [4]. More recently, other advanced catheter-based treatments including mechanical thrombectomy (MT) and aspiration thrombectomy (AT) have been studied for the treatment of upper extremity DVT as an alternative to CDT [5, 6]. After DVT removal, definitive treatment requires surgical decompression to resolve the underlying pathology, as recurrence of symptoms and loss of patency occurs in approximately half of patients treated with anticoagulation and CDT alone [7].

The ClotTriever System (Inari Medical, Irvine, CA) is a new MT system used for the treatment of DVT, approved by the US FDA and CE Mark in 2017. In the last few years, it has been used for lower and upper DVT mostly due to intravascular invasion or external compression from a known malignancy [8]. To the best of our knowledge, this is the first report on use of Inari ClotTriever for acute upper extremity deep venous thrombosis due to venous thoracic outlet syndrome. This report describes a young patient who underwent MT with the ClotTriever System for acute SV thrombosis in the setting of VTOS.

## Case report

A 29-year-old male presented to the emergency department for left upper extremity swelling, heaviness, limb fatigue with minimal exertion, and pain. He reported leading an active lifestyle and practicing cross-country skiing. Physical examination of the left upper extremity revealed swelling, tenderness to palpation over the bicep, and prominent venous vasculature. No prior history of thrombosis was reported.

**Fig. 1:**
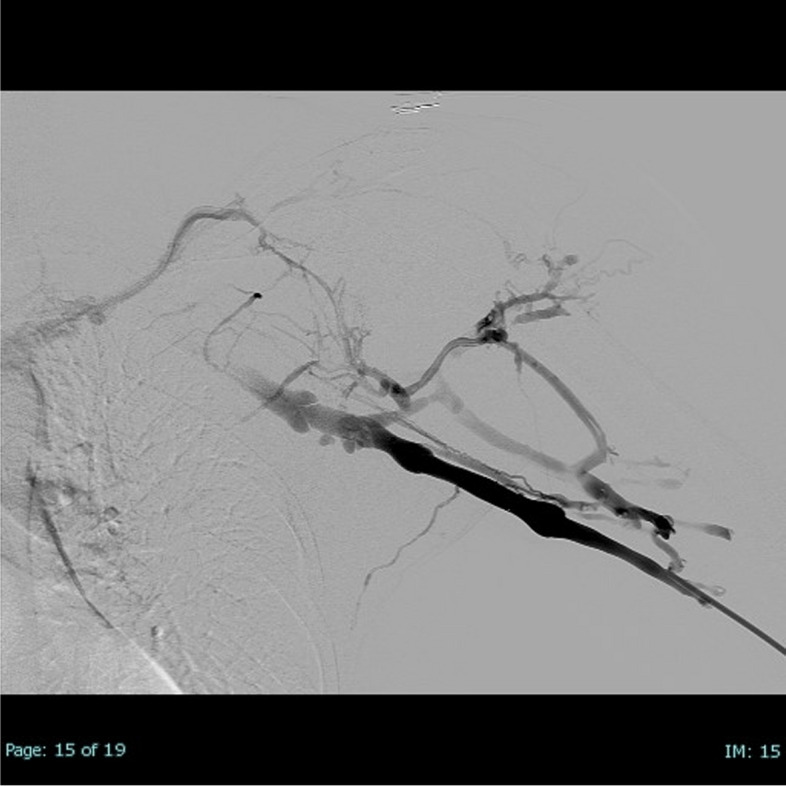
Initial venography confirms extensive thrombus from the axillary vein through the subclavian vein

Laboratory studies revealed hemoglobin of 15.9 g/dL, platelet count of 213,000/mm^3^, and an international normalized ratio of 1.09, thus ruling out coagulation disorders. Doppler Ultrasound (DUS) demonstrated a left SV thrombosis confirmed by computed tomography angiography of the chest (Fig. 1), which was negative for extension into the superior vena cava or left jugular vein. No pulmonary embolism or external regional masses were identified.

The patient was diagnosed with acute DVT of the left SV attributed to effort-induced thrombosis. Anticoagulation with low-molecular-weight heparin (LMWH) was initiated. After four days of treatment, the patient remained symptomatic with tenderness and swelling of the left upper extremity. MT with the ClotTriever System was proposed to and agreed upon by the patient.

Ultrasound-guided access of the left basilic vein was achieved with a micro-puncture technique. Extensive thrombus was noted on venography, extending from the axillary vein through the SV; thus, a 0.035-inch 260-cm Glidewire (Terumo, Japan) was advanced past the thrombus into the inferior vena cava. Following dilation, the 13F ClotTriever sheath with integrated thrombus capture funnel was inserted. The ClotTriever catheter was inserted through the sheath and positioned beyond the thrombus, the catheter coring element and an integrated collection bag were deployed. The coring element that separates thrombus from the vessel wall and the bag collects thrombus as the catheter is pulled back toward the sheath. An image of the deployed ClotTriever catheter in subclavian vein is provided in the supplementary materials. In this procedure, for the entire thrombus to be extracted, it was imperative to advance the device to the superior vena cava.

**Fig. 2:**
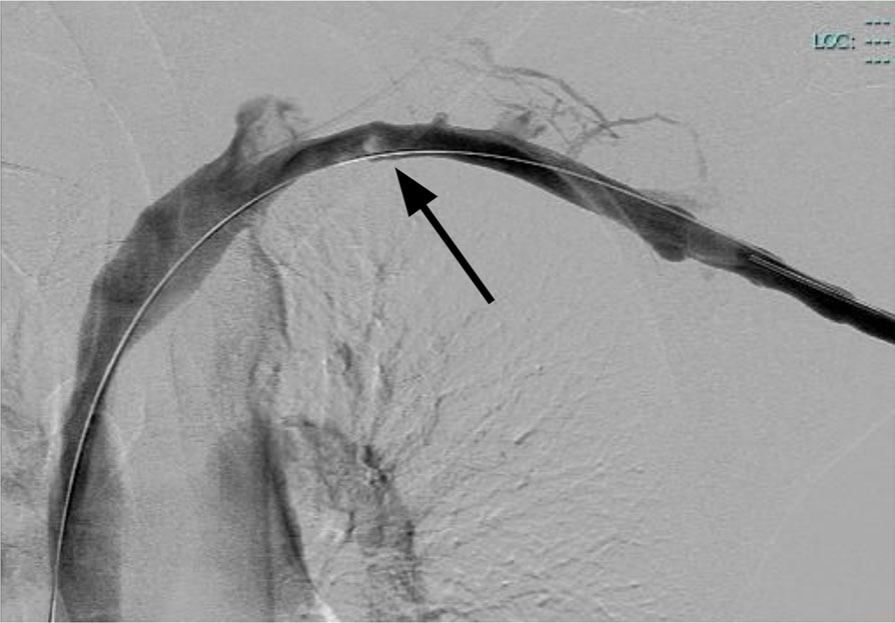
Final post-procedural venogram confirming > 90% reduction in thrombus burden (black arrow) with no-flow limiting fibrotic residue adhered to a valve flap on subclavian vein at the costoclavicular space

Successful MT was achieved after 4 ClotTriever catheter passes with a large amount of thrombus removed on the first pass. Comparison of the pre- and post-procedural venograms before indicated > 90% reduction in thrombus burden. Venography and intravascular ultrasound examination demonstrated the persistence of a no-flow limiting fibrotic residue adhered to a valve flap on SV at the costoclavicular space (Fig. 2), but no wall injuries. No procedural complications occurred and no significant arrhythmias were observed during the procedure. The procedure lasted 30 min, of which 6 min of fluoroscopy. The total dose area product was 0.0032 Gy/m^2^ and Air-Kerma was 0.1038 Gy. A procedural summary video is provided in the Supplementary Materials of this case report.

At the end of the procedure, the patient felt immediate relief in the upper extremity with a subsequent decrease in arm swelling. The patient was discharged to home later the same day with instruction to continue the LMWH therapy.

**Fig. 3:**
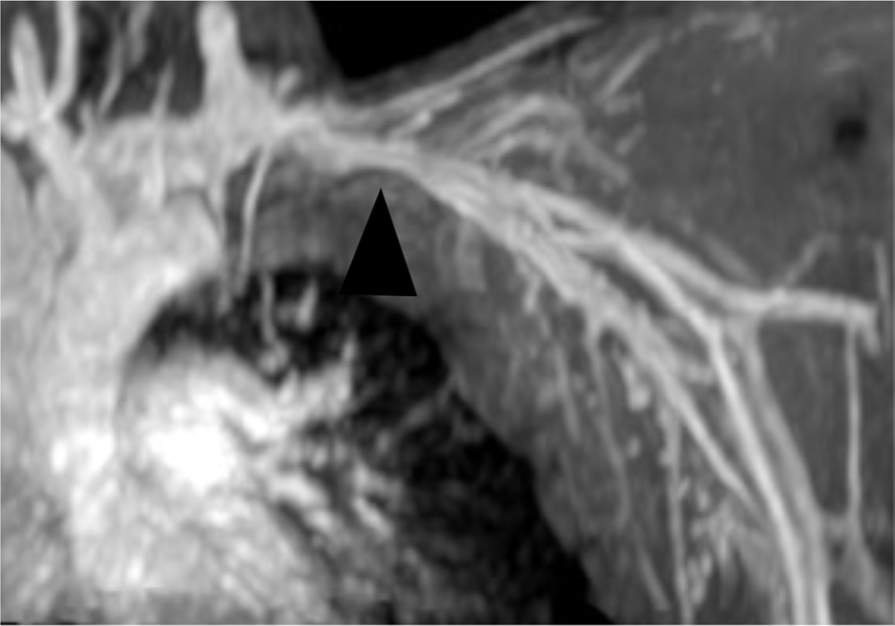
Follow-up magnetic resonance imaging scan conducted at the 3-month post-procedural visit demonstrating regular SV patency (black arrowhead)

At the 48-h follow-up visit, the patient's left upper extremity swelling and pain were fully resolved. Swelling was assessed by comparing the diameter of the affected arm at 48 h to the pre-procedural measurement (Axillary diameter before/after procedure 30 vs 28 cm, middle arm diameter 31 vs 27 cm, elbow diameter 30 vs 26 cm) and the diameter of the contralateral arm (axillary diameter 27 cm, middle arm 28 cm, elbow diameter 26 cm). One week after the procedure, he had regained full function of his upper extremity without limitations. At this time, LMWH was discontinued and substituted with Apixaban 5 mg twice daily. At the 3-month post-procedural visit the left upper extremity vein and internal jugular vein patency was confirmed with DUS and magnetic resonance imaging (Fig. 3).

Nine months later he underwent resection of the first rib to resolve the anatomical problem.

Written informed consent was obtained from the patient for publication of this case report and any accompanying images.

## Discussion

We successfully performed MT of the SV using the ClotTriever System for a patient with an effort-induced upper extremity DVT with confirmed venous patency and lack of complications through 3 months.

In the setting of VTOS, treatment of DVT with anticoagulation therapy alone may result in a residual chronic disability, such as post-thrombotic syndrome (PTS) [4]. Studies of CDT for the treatment of upper extremity DVT have previously reported successful restoration of patency following treatment, with one study suggesting low incidence of PTS [9]. Studies with pharmacomechanical CDT have also reported positive outcomes, one of which also reported decreased overall treatment time and tPA exposure with this approach compared to traditional CDT, however, major bleeding events were reported in both treatment arms [10]. More recently, studies of non-thrombolytic catheter-based treatments such as AT and MT have reported positive outcomes in the treatment of UEDVT [5, 6, 11].

In this case, the persistence of a mild residual stenosis at the costoclavicular space is not due to residual clot, but represents the fibrotic remodeling caused by intramural and extramural repetitive mechanical trauma on the SV.

## Conclusions

Although further studies are needed to clarify long-term patient outcomes and indications, the ClotTriever System may represent a new technique for thrombectomy in effort-induced thrombosis associated with VTOS.

### Supplementary Information

**Additional file 1: Media 1.** Procedure video summary.

## Data Availability

Data sharing is not applicable to this article as no datasets were generated or analysed during the current study.

## Abbreviations

VTOS: Venous thoracic outlet syndrome
SV: Subclavian vein
DVT: Deep vein thrombosis
CDT: Catheter-directed thrombolysis
MT: Mechanical thrombectomy
AT: Aspiration thrombectomy
DUS: Doppler Ultrasound
LMWH: Low-molecular-weight heparin
PTS: Post-thrombotic syndrome
